# Assessment of Patient-Specific Surgery Effect Based on Weighted Estimation and Propensity Scoring in the Re-Analysis of the Sciatica Trial

**DOI:** 10.1371/journal.pone.0111325

**Published:** 2014-10-29

**Authors:** Bart J. A. Mertens, Wilco C. H. Jacobs, Ronald Brand, Wilco C. Peul

**Affiliations:** 1 Department of Medical Statistics, Leiden University Medical Center, Leiden, The Netherlands; 2 Department of Neurosurgery, Leiden University Medical Center, Leiden, The Netherlands; 3 Medisch Centrum Haaglanden, The Hague, The Netherlands; Chinese Academy of Science, China

## Abstract

We consider a re-analysis of the wait-and-see (control) arm of a recent clinical trial on sciatica. While the original randomised trial was designed to evaluate the public policy effect of a conservative wait-and-see approach versus early surgery, we investigate the impact of surgery at the individual patient level in a re-analysis of the wait-and-see group data. Both marginal structural model re-weighted estimates as well as propensity score adjusted analyses are presented. Results indicate that patients with high propensity to receive surgery may have beneficial effects at 2 years from delayed disc surgery.

## Introduction

Sciatica is a term used to denote a collection of pain symptoms which may have several causes related to compression or irritation of spinal nerve roots leading into the sciatic nerve. The pain itself is typically located in the lower back, with the predominant pain radiating out into the leg, usually in only one side of the body. Pain symptoms range from mild to severe. Especially with severe pain, the disease can be debilitating, having a profound impact on physical and social functioning and lead to long periods of absence from work. The most common and well-known cause is spinal disc herniation (90% of diagnosed cases [1]). The disease is relatively common and affects 5 to 10 patients per 10000 individuals in Western countries annually [2] with disease risk increasing with known factors such as age, height, mental stress, smoking and exposure to vibration. There is no conclusive evidence of an association between risk of sciatica and gender or physical fitness. Fortunately, prognosis on acute sciatica is generally good, with 60% of patients recovering within 3 months and a further 10% within 12 weeks up to a full year [1] [2] [3]. Up to 30% of patients will however continue to experience pain for one year or longer [3] [4]. Absenteeism from work causes lumbar-spine disorders, such as sciatica, to have profound economic effects. Treatment options may be categorized into either conservative approaches (such as staying active, analgesics, non-steroidal anti-inflammatory drugs, up to multidisciplinary treatment) or invasive intervention through disk surgery. There is considerable dispute internationally about both long-term value and appropriate timing of surgical intervention, although consensus does exist that surgery should only be attempted after an initial period of conservative treatment. Few clinical trials exist to compare surgery with conservative treatment. Those that are available show no significant differences after at least 4 years follow-up [5] or even for shorter follow-up periods of two years [6] [7] [8] [9]. These trials suffer from poor compliance rates in the surgery groups, particularly for the latter trial (60% only for two years follow-up). Debate about either appropriateness or timing of surgery are further fuelled by uncertainty about disease progression and potential recovery (in the early stages) of the disease. Similar uncertainty affects our inability to screen out those patients who will eventually experience a malign and prolonged period of pain from what is otherwise a likely benign condition for most patients. Furthermore there are substantial sociocultural differences between - and even geographic variation within - countries on treatment preferences and practices. These are also part due to differences in health care provision, insurance systems and legislation related to work absenteeism. Thus for example, both the Netherlands and the United States have relatively high surgery rates for sciatica compared to other developed countries [7] [10]. The Netherlands sciatica guideline suggested surgery after a 6 weeks period of non-operative care, while other countries waited 6 months. Surgical treatment rates for lumbar discogenic syndromes for the United States are 40% higher than in any other country and more than 5 times larger than similar rates in the United Kingdom [11]. Surgery rates also seem to depend strongly on the number of neurologic and orthopaedic surgeons available per capita and preferences for surgery in other pathologies [12]. We apply both propensity score-based estimation and inverse-probability-of-treatment weighted estimation (marginal structural models [13]) to high quality data obtained in the framework of a clinical trial. The objective of these analyses is to reduce bias incurred in estimating the *per-patient surgery effect*. Such analysis is beyond the scope of the intention-to-treat analysis of the original randomization which could only assess the *effect of treatment policy* (conservative wait-and-see versus early intervention – as opposed to the individual surgery effect). The bias referred to will occur as a consequence of confounding by predictors (such as pain scores or the changes therein) which affect both the decision to take surgery as well as the outcome at end of follow-up [13] [14] [15].

## Methods

### The Leiden sciatica randomized trial data

The trial randomized 283 patients to either a wait-and-see (control, n = 142) or early surgery (microdiscectomy, n = 141) group. The wait-and-see group patients had the option to have surgery at a later date after inclusion, if the natural course was not leading to the desired leg pain relief and recovery of function. The follow-up period consisted of 2 years with one visit at baseline (randomization) and 9 subsequent scheduled monitoring visits, with the last visit at end follow-up (2 years). Four scores - Roland and Morris Disability Questionnaire (RMDQ), modified for sciatica, the Likert score for global perceived recovery and two visual analogue scales (leg pain and low back pain) - were measured at baseline and at all subsequent visits to monitor the condition of patients. We will refer to these generically as ‘pain scores’. [Trial registry and the registration number information: Current Controlled Trials number, ISRCTN26872154. First published: Peul, W.C. et al. (2007) Surgery versus Prolonged Conservative Treatment for Sciatica. New England Journal of Medicine, 356, 22, 2245–2256.].

### Statistical analysis

We analyzed the control group data at 2 years follow-up for treatment effect on RMDQ score as primary outcome and Likert score as secondary outcome. Invasive treatment was defined as surgery carried out prior to end of follow-up versus no surgery during follow-up. We calculated the propensity-to-receive-surgery score for each patient in the wait-and-see arm, defined as the probability to receive surgery during the follow-up, from a pooled logistic regression model as described in [13] and File S1. We decided to restrict analysis to the control group for several reasons. The processes which govern treatment decisions are different between both arms. In the immediate-treatment arm, patients receive surgery shortly after enrolment by design. In the wait-and-see arm however, surgery is a random event which is itself a response to the patient’s disease recovery process. Propensity-based methods require the so-called “positivity requirement” which states that patients must have positive probability to receive treatment - or remain free of treatment - post-baseline, which is clearly violated for the early surgery arm of the trial. Likewise, the treatment times distributions are completely separated between both study arms and thus nearly perfectly confounded with treatment between both arms. Furthermore, the recovery process post-surgery may be different between both arms.

Predictor variables used in the propensity model were age, gender, height, weight and the four scores (RMDQ, Likert score and both VAS scores) measured *at baseline*. In addition, we included the changes for each pain score at each visit as compared to those at the baseline and the ‘lagged differences’ in all pain scores, up to treatment or end of follow-up at 2 years if no surgery occurred. The ‘lagged changes’ are defined as the change in each VAS pain score at any visit with respect to the score observed at the immediately preceding visit. Pain score measurements or changes in pain levels, after the surgery, were not used in the treatment-prediction model as this would lead to biased surgery effect estimates. The study protocol actively offered non-recovering patients surgery at the fifth visit at 6 months after randomization, if not yet taken. Therefore, an indicator variable to account for higher proportions of patients taking treatment at the fifth visit was also included in the model. In the remainder of the paper, we refer to the “treatment decision point” as the moment where surgery was applied - or end of follow-up if no surgery was taken.

### Inverse probability of surgery weighted regression estimate

Effect of surgery was estimated using weighted regression analysis (marginal structural model [13]) with RMDQ and Likert scores at two years as dependent variables and treatment indicator as independent variable, while adjusting for baseline confounders and using propensity scores at the last visit as an inverse weight on each patient’s outcome. We carried out two secondary alternative (confirmatory) analyses to calculate the treatment effect on the RMDQ at end follow-up, using the propensity scores.

### Stratification on propensity of surgery score

Patients tend to share similar past pain histories and risk factors between the treated and non-treated groups within strata defined by percentiles of the propensity scores. Therefore, we first calculated separate treatment effect estimates within strata and then combined these across strata, by proportionally weighting each estimate for the relative fractions of numbers of patients within each stratum. This provides a bias-reduced treatment effect estimate.

### Regression with propensity of surgery-by-treatment effect interaction

Second, because the stratification-based analysis indicated possible change in the patient-specific surgery effect as a patient’s propensity to receive surgery increases, we generalized the stratification approach by carrying out a regression of outcome on treatment, adjusting for propensity score and including an interaction term between the propensity and treatment indicator (see [14] for a description of the approach). Results from the primary and secondary regression analyses are presented as medians and 95% confidence intervals for the effect estimates, based on (non-parametric) bootstrapping procedures using 10000 bootstrap samples (see [16] and File S1 for a complete explanation of the approach). The bootstrapping approach is used to correct for possible conservatism in estimation of the effect standard deviations. Comparisons of continuous (predictor) variables between treatment groups are based on the two-sample t-test and associated confidence intervals and p-values. Evaluation of the dependence of continuously distributed outcomes (RMDQ and Likert at end follow-up) on predictors is based on univariate regression and the p-values and confidence intervals of the corresponding regression coefficient.

### Ethics Statement

This paper concerns the re-analysis of data from a previously carried out randomised trial. Medical ethics committees at the nine participating hospitals who carried out the original randomized trial approved the protocol and written informed consent was obtained from all patients. No further medical ethical review is needed for the secondary analyses reported here.

## Results

**Figure 1:**
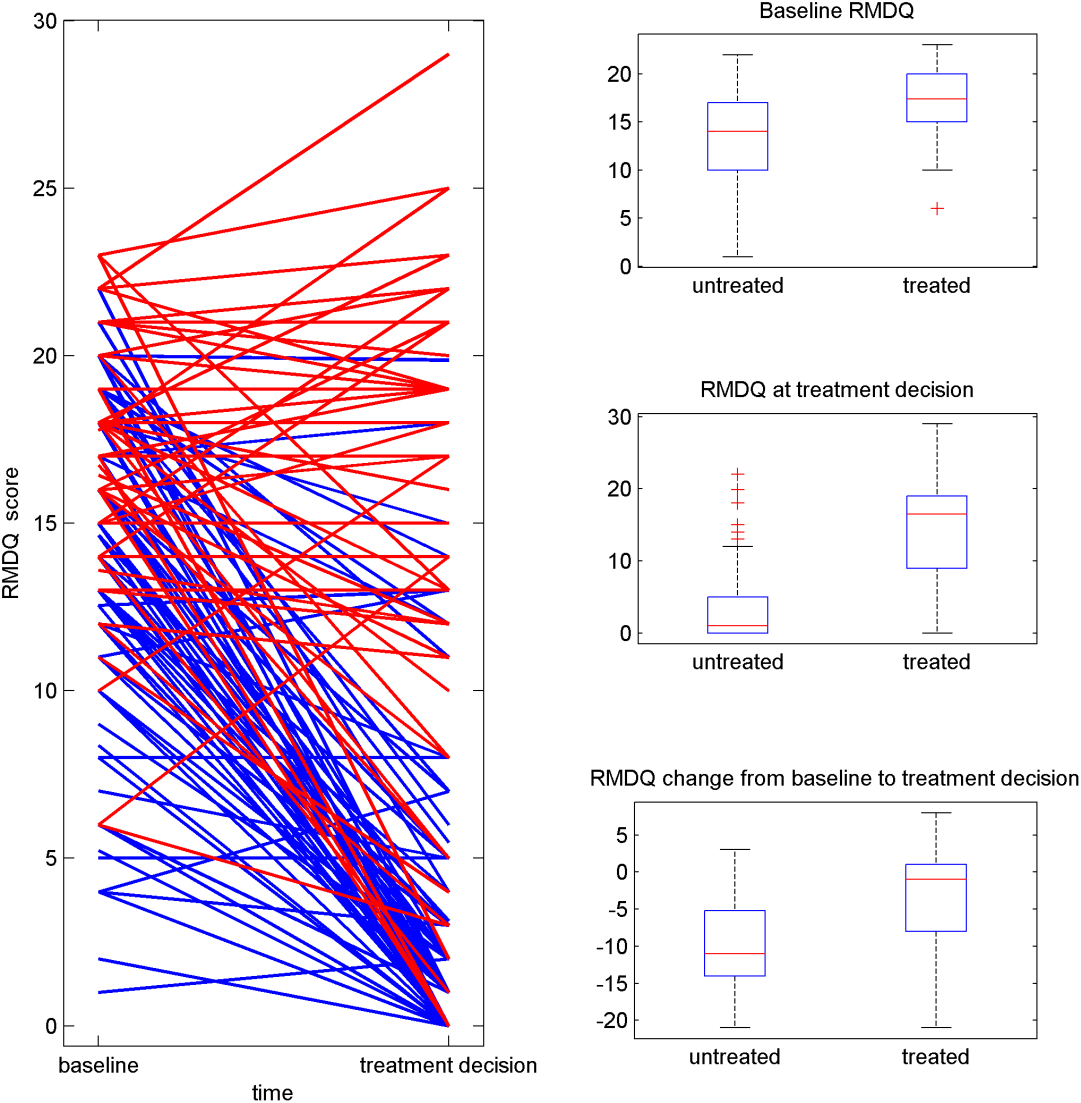
The left plot in Figure 1 shows RMDQ scores for each patient separately, plotted as a connected line for each patient between the baseline measured value (“baseline”) and the point immediately prior to surgery - or end of follow-up if no treatment was taken (“treatment decision”). Treated individuals are shown as red profiles while untreated individuals are represented with blue profiles. To the right, three boxplots are shown for treated and untreated individuals separately and for RMDQ scores at baseline (top), RMDQ scores at “treatment decision” (middle) and for the change in RMDQ score from baseline to treatment decision point (bottom).

The wait-and-see group contains 142 patients, approximately 40% (56) of which opted into surgery during follow-up, of whom 15 within two months from randomization, 37 between two months and one year, and four patients after one year. Table 1 gives 95% confidence intervals (CI) and p-values (P) for the differences between treatment groups (Treatment) as well as association with outcome at two years (RMDQ, Likert) for gender, weight, height, age and the baseline pain scores as well the changes in these scores prior to the treatment decision. Comparisons of baseline values between treated and untreated patients at 2 years indicate significantly worse condition for RMDQ (P<0.0001), Likert score (P = 0.014) and VAS leg pain score (P = 0.011), as higher baseline scores are observed for surgically treated patients compared to non-operative and marginally significant for VAS low back pain (P = 0.12). Comparison of the change from baseline between both treatment groups shows significant differences for all pain scores (RMDQ: P<0.0001, Likert score: P<0.0001, VAS leg pain: P<0.0001, VAS low back pain: P<0.0001) with smaller pain reductions from baseline for patients who decided to take treatment. Figure 1 shows a graphical illustration of the situation for the RMDQ scores between baseline and the treatment decision. Individuals who eventually opted for treatment are clearly identified as having higher baseline scores and their profiles after baseline tend to show smaller reductions or even increases in pain score - as opposed to individuals not taking surgery. We did not find substantial evidence of the (lagged) changes in pain scores immediately preceding the decision to treat (or the final follow-up measurement if the patient was untreated) affecting the treatment decision (RMDQ: P = 0.21, Likert score: P = 0.11, VAS leg pain: P = 0.20, VAS low back pain: P = 0.027). We found evidence that the baseline pain scores predict the observed levels at outcome for the RMDQ score (RMDQ: P = 0.018, Likert score: P = 0.023, VAS leg pain: P = 0.012, VAS low back pain: P = 0.0087) but not so for the Likert score (RMDQ: P = 0.85, Likert score: P = 0.92, VAS leg pain: P = 0.75, VAS low back pain: P = 0.46). Likewise, the changes in Roland score from baseline are predictive of the observed level of the same score at end of follow-up (P = 0.0011) and similarly for the Likert score (P = 0.0091). The lagged changes in Likert score between both visits immediately preceding the point of treatment (or end-of follow up if no treatment) was found to be predictive of the observed level of the same score at end of follow-up (P = 0.0013), but not so for the RMDQ score (P = 0.14). There is no evidence of strong predictive effects of age, gender, height or weight on either the decision to treat or on final outcome (table 1).

**Table 1:** Tabulation of predictors used in the treatment propensity model, distinguishing between baseline variables and variables subsequent to baseline.

	Treatment		Roland		Likert	
	CI	P-value	CI	P-value	CI	P-value
Baseline predictors						
age	(−4.23, 2.26)	0.55	(−0.04, 0.12)	0.28	(−0.00, 0.03)	0.07
gender	(−0.21, 0.11)	0.52	(−0.59, 2.68)	0.21	(−0.26, 0.48)	0.56
weight	(−1.19, 8.25)	0.14	(−0.08, 0.03)	0.43	(−0.01, 0.02)	0.39
height	(−2.88, 3.49)	0.85	(−0.15, 0.02)	0.12	(−0.01, 0.02)	0.68
VAS-1	**(2.47, 18.40)**	0.01	**(0.01, 0.07)**	0.01	(−0.01, 0.01)	0.76
VAS-2	(−1.90, 17.10)	0.12	**(0.01, 0.06)**	0.01	(−0.00, 0.01)	0.46
Roland	**(2.12, 5.19)**	0.00	**(0.03, 0.34)**	0.02	(−0.03, 0.04)	0.85
Likert	**(0.09, 0.79)**	0.01	**(0.12, 1.55)**	0.02	(−0.16, 0.17)	0.93
Changes from baseline						
VAS-1	**(17.16, 35.51)**	0.00	(−0.01, 0.04)	0.35	(−0.00, 0.01)	0.42
VAS-2	**(15.79, 34.65)**	0.00	(−0.02, 0.03)	0.80	(−0.01, 0.01)	0.96
Roland	**(4.69, 9.04)**	0.00	**(0.07, 0.27)**	0.00	(−0.02, 0.03)	0.60
Likert	**(0.72, 1.75)**	0.00	(−0.14, 0.79)	0.17	**(0.04, 0.24)**	0.01
Lagged changes						
VAS-1	(−7.15, 1.53)	0.21	(−0.07, 0.05)	0.71	(−0.01, 0.02)	0.38
VAS-2	**(0.78, 12.04)**	0.03	**(−0.10,** **−0.02)**	0.01	**(−0.03, −0.01)**	0.00
Roland	(−1.38, 0.30)	0.21	(−0.07, 0.53)	0.14	(−0.05, 0.09)	0.65
Likert	(−0.60, 0.07)	0.12	(−0.28, 1.24)	0.22	**(0.11, 0.45)**	0.00

‘Change from baseline’ refers to the difference between the baseline value of any pain score and the last measurement prior to the treatment decision, or end of follow-up if no treatment. ‘Lagged changes’ are the differences between the corresponding pain score measures at the last two visits prior to treatment, or end of follow-up. The ‘Treatment’ column gives 95% confidence intervals for mean differences between individuals treated and left untreated by end of the trial. The columns ‘Roland’ and ‘Likert’ give 95% confidence intervals for the regression coefficient of each of these two scores in a univariate regression on each predictor individually.

### Inverse probability of surgery weighted regression estimate

The median surgery effect estimate from inverse probability weighted regression analysis was −2.10 (95% CI −4.21, 0.07) for the RMDQ as compared to the median classical regression-based estimate of −1.47 (−2.72, −0.14) which is adjusted for the same confounders. For the Likert score, the median inverse probability weighted surgery effect estimate was −0.21 (−0.72, 0.24), as compared to the classical regression-adjusted estimate of −0.28 (−0.62, 0.05). Recent insight in propensity-score based estimation suggests that effect estimates and confidence levels particularly, may be affected by overfitting of the propensity score model [15] [17]. This may be due to a combination of a relatively small sample size and excess numbers of predictors, as well as high correlation between the treatment predictors, such as between baseline pain scores and the subsequent changes in these for the present analysis. We therefore decided to remove predictors in a stepwise fashion from the propensity score model. The deviance statistic (File S1) was calculated for each deletion to check no significant changes to model fit occurred. We first removed (1) the effects of gender, weight and height, then (2) the effect of age, (3) then the VAS leg pain and VAS low back pain lagged changes and finally (4) lagged changes and changes from baseline for the RMDQ score. Using the reduced set of predictors, we re-estimated the surgery effect on the RMDQ score with the new propensity model which gave a median treatment effect of −2.52 (−4.77, −0.16) as compared to the classical regression estimate of −1.31 (−2.48, −0.09) adjusting for the same set of confounders. For the Likert score, the corresponding median surgery effect estimate using the updated propensity score is −0.29 (−0.75, 0.16) as compared to the classical regression estimate of −0.26 (−0.58, 0.05). Since no substantial differences were found between the classical and reweighting based results for the Likert score and the effect estimates are consistently small and non-significant, we decided not to investigate the Likert outcome further. For the Roland score, we carried out an additional classical regression of the outcome on treatment indicator which also adjusts for both propensity score and all baseline variables. The median surgery effect estimate from 10000 bootstrap repetitions of this procedure was −1.67 (−3.17, −0.12), confirming from a qualitative point of view the previous results but with a more narrow confidence interval. We then decided to carry out a subsequent confirmatory exploratory analysis on this outcome.

### Stratification on propensity of surgery score

**Figure 2:**
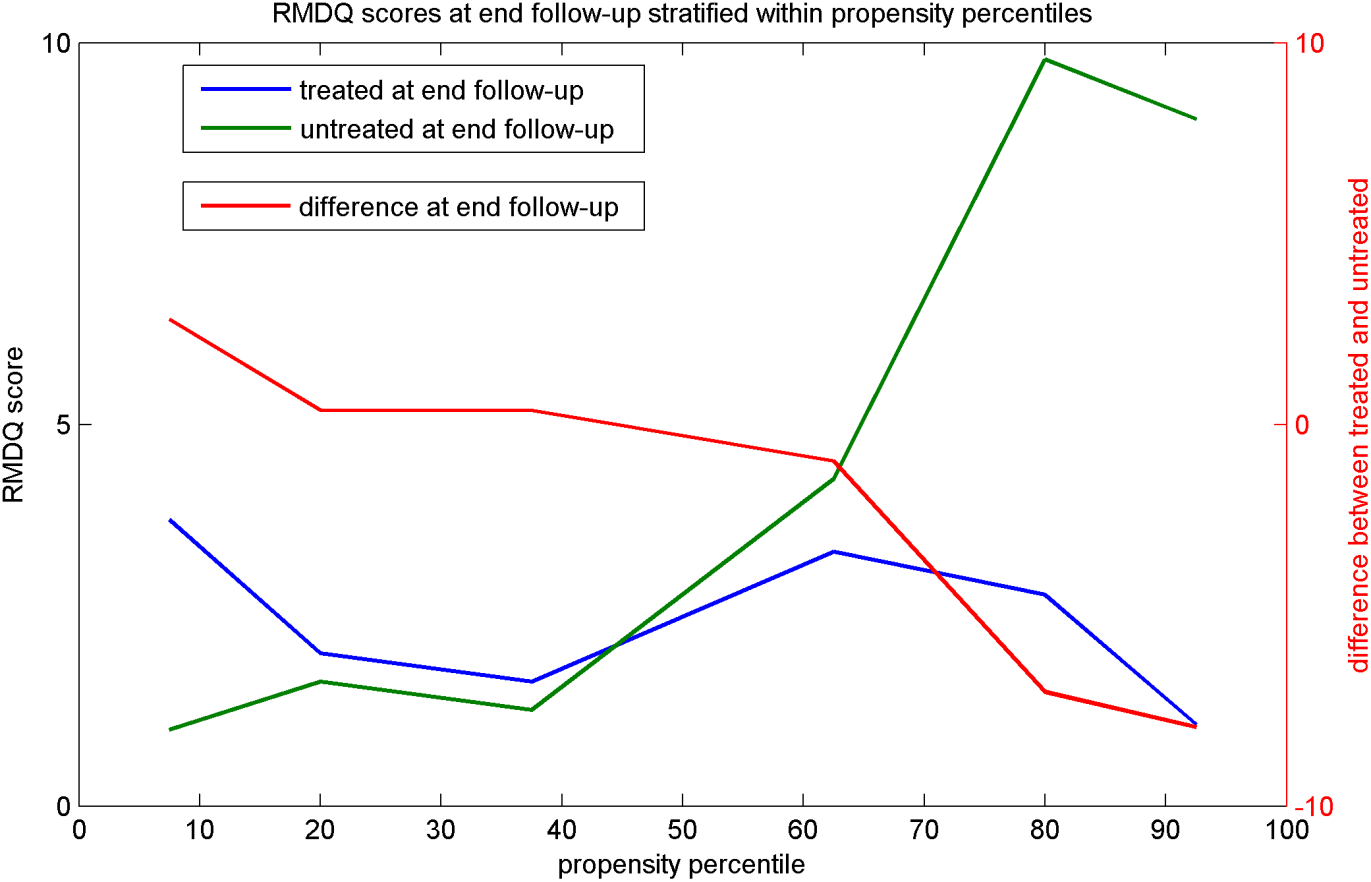
Figure 2 shows mean RMDQ scores within propensity percentiles strata as defined in table 2, plotted versus the midpoint of each percentile stratum and for treated and untreated patients separately (left-side axis). The red curve shows the difference in RMDQ scores between the treated and untreated patients at end follow-up across propensity percentile strata (right-side axis).

Table 2 shows stratified mean RMDQ scores at baseline and at end of follow-up for both treatment groups and across 6 strata of the propensity score, defined by cut-offs at the 15th, 25th, 50th, 75th and 85th percentile of the propensity score. As in table 1, we find that smaller propensity to have surgery is associated with lower baseline scores. RMDQ scores are slightly elevated in the delayed surgery group as compared to non-operated individuals at baseline within each stratum, indicating a small residual imbalance after stratification. While the latter effect disappears at end follow-up, an upward trend is apparent in mean RMDQ score levels at end of follow-up for untreated individuals, particularly for the last two propensity strata where patients maintain high expected mean RMDQ scores (9.78 and 9.00 for the 75–85% and 85% strata). Such trend is not observed for surgically treated individuals at end follow-up, whose mean scores are all lower than 3.3 with no clear pattern. The per-stratum treatment effect estimates (last column ‘difference’), defined as the differences between mean RMDQ scores between surgically treated and untreated individuals at end follow-up, show clear inhomogeneity of effect and a downward trend as the propensity to receive surgery increases (i.e. ever greater reductions of the RMDQ score at end follow-up). The latter effect appears predominantly due to the above described tendency for RMDQ scores to remain at high levels for untreated individuals with high treatment propensity. This trend in effect estimates is however also affected by the fact that mean RMDQ outcomes remain larger for treated individuals as compared to untreated individuals across the first 3 propensity strata. Figure 2 shows a graphical representation of the same information. The sequence of surgery effect estimates in the last column of table 2 (figure 2) has a strict monotone decreasing order, with the effect estimates actually changing sign from an increase in mean RMDQ score of 1.75 for patients at smallest propensity (due to a relatively good condition at outset of the trial) up to decreases in RMDQ scores of up to −7.93 for patients with the highest surgery propensity. The average weighted surgery effect of −1.74 which combines the stratum-specific effect estimates is in line with estimates from the primary analysis. This estimate, however, masks substantial change of the effect of surgery as the condition of the patient - and thus also the propensity to take the treatment - changes.

**Table 2:** Results for Roland scores stratified for percentiles of propensity scores.

propensity percentile	Baseline		end follow-up (outcome)		difference at end follow-up
	untreated	treated	untreated	treated	
<15%	8.11	10.75	1.00	3.75	1.75
15%–25%	11.63	17.33	1.63	2.00	0.38
25%–50%	14.03	16.55	1.26	1.63	0.36
50%–75%	15.86	15.86	4.29	3.33	−0.96
75%–85%	16.30	18.13	9.78	2.77	−7.01
>85%	15.33	18.00	9.00	1.07	−7.93
weighted average of surgery effect across strata at end follow-up					−1.74

The first column shows the percentile intervals of propensity score. Subsequent columns show mean Roland scores within each percentile interval and for treated and untreated individuals at baseline and for the outcome at end of follow-up. The last column shows differences between mean Roland outcomes for treated and untreated individuals.

### Regression with propensity of surgery-by-treatment effect interaction

To account for possible heterogeneity of treatment effect, we estimated a regression model of RMDQ on treatment, adjusting for propensity score, all baseline variables and a propensity-by-treatment interaction effect (see [14] for a similar procedure). The median treatment-by-propensity effect from this model, based on 10000 bootstrapping repetitions was −29.23 (−99.85, −8.80). This estimate implies a median reduction in VAS pain score of −0.90 (−7.59, 2.68) for individuals at median propensity (0.065) while individuals at the 75th percentile of propensity would have median VAS pain score reductions of −4.85 (−21.07, 1.49). Individuals at the 25th percentile would experience median increases in VAS pain of 0.11 (−4.10, 2.97).

## Discussion

The sciatica randomised trial is an assessment of the effect of policy (early intervention or a wait-and-see approach) *at the population level*. We have shown that within the wait-and-see arm of the trial, the decision to take surgery is associated with heightened pain scores at baseline and smaller reductions in those scores after baseline as compared to patients who remain free of surgery up to the end of the trial. The sciatica trial found no evidence of significant differences in RMDQ scores at either 1 or 2 years of follow-up between both trial arms. Because of confounding of the treatment decision with past pain score history within the wait-and-see arm, such results from the original trial should however not be (mis) interpreted in terms of lack of potential effect at the *individual patient level*. Use of inverse-probability-of-treatment weighted estimation and propensity scores in statistical analysis can reduce bias incurred in estimating the *per-patient surgery effect*, due to treatment predictors being confounded between pain scores at outcome and the treatment decision [14]. Re-weighting methods reduce bias by decreasing the influence of patients who are over-represented in the surgery group at end of trial, because of their heightened probability to receive surgery as a consequence of confounders, such as a relatively poorer condition at outset or relatively greater deterioration of the patient’s state during the observation period. Such variation in individual propensity is of course completely understandable and natural, but cannot be accounted for with classical analysis methods in RCT intent-to-treat analysis, when we are interested to uncover the surgery effect itself. Likewise, patients with small probabilities of receiving treatment in the group which remains free of surgery by end of trial are given higher weight, for similar reasons.

Propensity score-based approaches to treatment effect estimation solve the non-random treatment allocation based on past pain levels, by exploiting the balancing property that patients who share the same level of treatment propensity tend to have similar levels of the confounding variables.

Our application of both re-weighting and propensity-based estimation suggests that patients with markedly poor baseline condition with regard to pain and function and who do not experience sufficient improvement in condition may benefit from surgery intervention, as the decision to take treatment is strongly dependent on such pain history. Patients at the smallest levels of propensity may however be adversely affected by surgery.

These results suggest prediction methods may be devised to screen out patients most likely to benefit from surgery. Unfortunately, the propensity score cannot be used for this purpose - at baseline at least - as it depends on future observations not yet available at the baseline. Propensity scores are only retrospectively derived for the purpose of identifying individuals who are comparable with respect to potential confounders to allow for an assessment of treatment effect, after the data has been collected. Another problem with the propensity-based methodologies presented in this paper is that they are strongly dependent on all confounders between the treatment decision and the outcome pain scores to be known and available. Since our analysis is concerned with the secondary analysis of a randomised trial, this requirement may not be met. It would be helpful - even for randomized trials generally - where treatment decision may depend on prior information to record such information as accurately as possible in order to facilitate such secondary analysis as we have described in this paper, even if this is not the primary purpose of the trial. Likewise, it would be helpful if greater care could be applied in the discussion of randomised trial results to make a careful distinction between effect interpretations at either the population or patient-specific level - as and when required - as for the present trial.

The current paper does not address the issue of validation of research findings presented here. Validation could be attempted in several ways - through comparison with other similar studies - or through some form of predictive validation on new patients - both of which would however raise substantial methodological challenges – and require extensive novel data analytic work. Both of these are beyond the scope of the present paper. A recent publication [18] for example, could provide data of a similar nature as described in this paper. Application of the methodologies described in this paper to that study’s data would however involve calibrating from scratch a suitable propensity score based on that study’s data, as it is inherent in propensity score methodology that the score must be calculated separately for each study. At the time of writing, the purely methodological issues involved in causal inference comparative studies are effectively unexplored. Similarly, research is emerging on application of causal effect estimation approaches to predict future patient’s potential outcomes. Application of this in clinical research is currently however largely unexplored. Readers should beware these issues place restriction on the interpretation of findings presented here.

We should note that this RCT, as well as others, excluded patients in a fairly good state or those predominantly complaining about low back pain instead of leg pain. This results in a lack of contrast with care-as-usual and therefore one has to be careful with generalizing our results to guidelines. However, the results of this propensity-based analysis, in combination with the lack of contrast in trial circumstances as compared to realistic daily care, suggests future studies may be designed to devise prediction methods with a higher predictive value of RMDQ and VAS leg-pain scores than currently considered possible. This provides further argument to the need for careful and well thought through registration in future trials, specifically to record all indicators for surgery - at baseline - but also subsequently in the trial when patients receive surgery post baseline, as well as the duration of sciatica and time to surgery. Such studies may finally deliver medical science the screening methods capable of identifying the most acute cases who are likely to benefit from surgery, while protecting individuals whose condition would unlikely improve due to surgery from unnecessary, costly and potentially harmful intervention.

## Supporting Information

File S1 **Details of statistical analyses presented in the paper.**
(PDF)
