# Supplementary material for: Assessment of Patient-Specific Surgery Effect Based on Weighted Estimation and Propensity Scoring in the Re-Analysis of the Sciatica Trial
Source: PLoS One. 2014 Oct 29;9(10):e111325. doi: 10.1371/journal.pone.0111325 (PMC4213022; doi:10.1371/journal.pone.0111325)
Supplement: File S1 — Details of statistical analyses presented in the paper. (PDF) [file pone.0111325.s001.pdf]

S1 - Supplementary material to: Assessment of patient-specific surgery effect based on weighted estimation and propensity scoring in the re-analysis of the Sciatica Trial.

Mertens, B. J. A.[1]*, Jacobs[2], W.C.H., Brand[1], R., and Peul[2], W.

October 3, 2014

Departments of Medical Statistics[1] and Neurosurgery[2], Leiden University Medical Center, PO Box 9600, 2300 RC Leiden, Netherlands

1 Inverse probability treatment weighted analysis

Marginal structural models are estimated in two stages. At the first stage, a treatment prediction model is fit, which is used to calculate the patient-specific probabilities to receive treatment (surgery in this case) at any point in time while the patient has not yet received the treatment. At the second stage, the inverses of these treatment probabilities are used as weights in the calibration of the substantive model, such as a regression model or other on

*Correspondence to: Bart J. A. Mertens, b.mertens@lumc.nl, http://www.lumc.nl.
This research received no specific grant from any funding agency in the public, commercial, or not-for-profit sectors.

the outcome of interest, which is used for the assessment of the treatment effect.

For the fitting of the treatment prediction model we may only use the observed data up to and including the time of surgery, or end of follow up, whichever comes sooner. All data subsequent to the surgery is discarded. Let $k \in \{1, ..., 9\}$ denote the visit numbers in follow-up and $k = 0$ denotes the baseline. Then we write $L(i, t_{ik})$ for the set of all observed scores (VAS1, VAS2, Roland and Likert) for any i^{th} patient at the kth visit, with corresponding visit time t_{ik} after baseline. A patient who remains free of surgery till end follow-up will contribute the full set of measurements $\{L(i, t_{i1}), ..., L(i, t_{i9})\}$ across all visits to the fitting of the treatment prediction model. If an ith patient receives surgery at time t_{is} and there are $k - 1$ scheduled visits prior to surgery for this patient, then the patient contributes the set $\{L(i, t_{i1}), ..., L(i, t_{i(k-1)}), L(i, t_{is})\}$ to the data used for fitting the weights model. Should surgery take place prior to the next scheduled visit, then we define the visit number corresponding to the final measurement to be $k(t_{is}) = k$. In addition, we have for each ith patient the baseline set of covariates $V(i)$, which consists of the above mentioned four baseline scores $L(i, 0)$, besides age, gender, height and weight. In analogy to the above, we define an additional set $M(i, t_{ik})$ of time-dependent measures on visits $k > 0$ defined as the changes in the VAS1, VAS2, Roland and Likert scores from baseline at each time point as well as the changes in these scores from each previous time point (lag values). Finally, we may define the treatment (surgery) indicators $A(i, t_i)$ for any patient such that $A(i, t_i) = 1$ for any time point from surgery onwards ($t_i \geq t_{is}$) and zero otherwise or when surgery never took place for that patient. Note $A(i, 0) = 0$ by definition and

that treatment can only be given once.

1.1 Treatment probability prediction model for IPTW weighting

Calibration of a treatment probability model is formally a survival analysis problem on time till treatment and thus Cox regression could be used. Hernan, Brumback and Robins [1][2][3][4] propose pooled logistic regression models for the estimation of the treatment probabilities. While the main arguments seem to be simplicities of the approach and ease of implementation, we refer readers to the above cited papers for full discussion of the pros and cons involved. To remain consistent with these publications, we decided to apply the same methodology on the sciatica data in this paper. Let $P_{i,t}$ be the treatment probability for the ith patient at time t and conditional on the patient not yet having received treatment at that point. We then formulate the model as

$$\log\left(\frac{P_{i,t}}{1-P_{i,t}}\right) = \alpha + h(t) + [L(i,t)\boldsymbol{\beta}_L] + [M(i,t)\boldsymbol{\beta}_M] + [V(i)\boldsymbol{\beta}_V]. \qquad (1)$$

The term $[L(i,t)\boldsymbol{\beta}_L]$ is a shorthand notation for the linear combination of the scores within the predictor set $L(i,t)$ with regression coefficients in the vector $\boldsymbol{\beta}_L$ and similarly for the baseline component $[V(i)\boldsymbol{\beta}_V]$ and for $[M(i,t)\boldsymbol{\beta}_M]$. The function $h(t)$ is a function of time since randomization only and defined as

$$h(t) = \gamma t + \alpha I(k(t) = 5),$$

where I() denotes the indicator function having a value of 1 when its argument is true and 0 otherwise and $k(t)$ denotes the visit interval number which corresponds to time t. The last term represents a probability spike at the fifth visit. The latter is to account for a sudden increase in surgeries carried out as a consequence of the trial actively offering the surgery to patients who had not yet received it at that stage and did not show recovery of function or pain. We carried out a confirmatory analysis on our choice of the time component by starting with the more general model

$$h(t) = \gamma t + \sum_{k=1}^{9} \alpha_k I(k(t) = k)$$

and then incrementally merging time points and their coefficients α_k from the last visit backwards or from the first visit onwards. By checking deviances we found that all time points could indeed be merged within the model, except for the fifth visit. While this approach is simple, it is however essential to enforce parsimony on the calibration of the weights as much as possible, because inverse probability weighted effect estimation could be highly sensitive to excess variation of the weights (see subsequent results and discussion).

1.2 Inverse probability treatment weights

Once the treatment probabilities $P_{i,t}$ have been calibrated at the observed visit and treatment times, the estimated weight $W(i)$ for any i^{th} person are defined as

$$W(i) = \frac{W_{num}(i)}{W_{denom}(i)} \quad (2)$$

with the denominators defined as

$$W_{denom}(i) = P_{i,t_{is}} \prod_{l=1}^{k-1}(1 - P_{i,t_{il}}) \tag{3}$$

if the individual did have treatment at time t_{is}. Otherwise we have

$$W_{denom}(i) = \prod_{l=1}^{9}(1 - P_{i,t_{il}}) \tag{4}$$

for those people never taking treatment up to end of the trial. The numerators $W_{num}(i)$ are stabilizing weights calculated in the same manner from complementary logistic models to the above described. They have identical definition as the above model, except that all the time-dependent predictors are removed from the model (keeping only the above defined 8 baseline predictors and the time component). An inverse probability of treatment weighting approach then applies these weights to the likelihood as in

$$\prod_{i=1}^{n}[lik_i]^{W(i)},$$

where the lik_i are the likelihood components for each ith patient (see also [1], page 442).

Tables 1 and 2 show median of bootstrap effect estimates as well as a 95% confidence interval from both an inverse treatment probability weighted analysis (IPTW, first 3 columns) as well as a classical unweighted analysis (next 3 columns, adjusted for baseline variables only) of the above covariance regression model for Roland and Likert scores respectively, based on 10000 bootstrapping repetitions, to account for the variability of the weight esti-

mation. The top row in the table ('all preds') gives results when including all predictors.

The weights model may leave unknown confounders unaccounted for. Nevertheless, the model may include too many predictors, particularly as these are highly correlated (such as the baseline and subsequent change from baseline measures) and thus may inflate the variance of weight prediction, which is undesirable. Some balance must be struck between the need to enforce parsimony of weight estimation as much as possible with capturing the required confounding information in the weight prediction. We address the problem by successively reducing the weight prediction model by removing predictors and then re-calculating the weighted estimates, removing the change scores last. The results from this procedure are shown in subsequent rows to the first where predictors are removed from the model as shown in the last column. For consistency, we always remove the same predictors from the covariance regression models (both IPTW and Classic). The last column in both tables shows deviances from the logistic regression weight model 1 on treatment probability, which give an indication of the impact of removing predictors on the change in calibrated weights relative to the previous model.

Table 1: Median of bootstrap estimates and 95% confidence intervals based on 10000 bootstrap replications for the treatment effect on Roland scores at end of study, based on application of IPTW weighted analysis (columns 1-3) as well as unweighted analysis (classic, columns 4-6) of a covariance adjusted regression of Roland score at end of study on treatment indicator, adjusted for baseline covariables. A marginal weighted estimate is also presented (columns 7-9). The analysis is repeated in subsequent rows to the first by removing predictors from the treatment probability prediction model (column 10). Deviances of each treatment probability prediction model are shown in the last column.

IPTW			Classic			Marginal weighted				
2.5%	med	97.5%	2.5%	med	97.5%	2.5%	med	97.5%	Model	Dev
−4.21	-2.10	0.07	−2.72	-1.47	−0.14	−2.94	-1.39	0.42	all preds	315.50
−4.74	-2.40	0.01	−2.42	-1.25	−0.02	−3.44	-1.66	0.20	-gender, weight, height	318.14
−5.07	-2.49	0.06	−2.51	-1.32	−0.07	−3.59	-1.69	0.23	-age	318.35
−5.07	-2.52	−0.03	−2.46	-1.32	−0.09	−3.61	-1.74	0.21	-VAS1/2 lags	323.35
−4.77	-2.51	−0.16	−2.48	-1.31	−0.09	−3.42	-1.72	−0.08	-Roland (chg/lags)	325.32
−4.23	-2.17	−0.19	−2.50	-1.31	−0.11	−3.06	-1.61	−0.12	-Likert (chg/lags)	340.40
−2.47	-1.32	−0.09	−2.47	-1.32	−0.09	−1.83	-0.84	0.14	-VAS1/2 chg	374.98

Table 2: Median of bootstrap estimates and 95% confidence intervals based on 10000 bootstrap replications for the treatment effect on Likert scores at end of study, based on application of IPTW weighted analysis (columns 1-3) as well as unweighted analysis (classic, columns 4-6) of a covariance adjusted regression of Likert score at end of study on treatment indicator, adjusted for baseline covariables. A marginal weighted estimate is also presented (columns 7-9). The analysis is repeated in subsequent rows to the first by removing predictors from the treatment probability prediction model (column 10). Deviances of each treatment probability prediction model are shown in the last column.

IPTW			Classic			Marginal weighted				
2.5%	med	97.5%	2.5%	med	97.5%	2.5%	med	97.5%	Model	Dev
−0.72	-0.21	0.24	−0.62	-0.28	0.05	−0.74	-0.24	0.23	all preds	315.50
−0.69	-0.20	0.25	−0.55	-0.24	0.06	−0.70	-0.23	0.22	-gender, weight, height	318.14
−0.68	-0.19	0.28	−0.57	-0.26	0.05	−0.70	-0.22	0.24	-age	318.35
−0.70	-0.23	0.23	−0.57	-0.26	0.05	−0.68	-0.26	0.16	-VAS1/2 lags	323.35
−0.75	-0.29	0.16	−0.58	-0.26	0.05	−0.72	-0.33	0.07	-Roland (chg/lags)	325.32
−0.73	-0.29	0.15	−0.57	-0.26	0.05	−0.74	-0.36	0.06	-Likert (chg/lags)	340.40
−0.58	-0.26	0.05	−0.58	-0.26	0.05	−0.54	-0.25	0.03	- VAS1/2 chg	374.98

1.3 Marginal weighted analysis

For comparison, we may consider a marginal analysis, using the new weights

$$W_m(i) = \frac{\frac{1}{W_{denom}(i)}}{\sum_{j=1}^{n} \frac{1}{W_{denom}(j)}}, \quad (5)$$

where the $W_{denom}(i)$ are defined as before and use the same baseline and time-dependent covariates and the sum is over all individuals. We can now define a weighted mean difference in an outcome measure X (such as $L(t_9)$ in this paper) as

$$\Delta = \sum_{i=1}^{n} W_m(i)X(i)I(T(i) = 0) - \sum_{i=1}^{n} W_m(i)X(i)I(T(i) = 1). \quad (6)$$

A (bootstrapped) standardized version of Δ can be considered an analogue of a t-statistic. See the tables for these results.

1.4 Propensity scores for stratification

We define the propensity scores $P(i)$ as the probability of treatment for each ith patient, based on model 1 with all predictors added, but now only retaining either the last calculated probability of treatment $P_{i,t_{is}}$ from the model if the person has treatment at time t_{is} and otherwise $P_{i,t_{i9}}$ for untreated individuals at the end of the trial (last visit = 9). The corresponding estimates are simply obtained from the fit of the full pooled logistic model discussed before for the MSM approach, hence no new calculations are required.

1.5 Regression model with propensity scores

Adjusted estimates of treatment effect are obtained by adding the propensity scores and baseline variables as covariables to a simple linear regression model on the final observed outcome scores at visit 9, besides the treatment indicator. So the final regression model for the propensity-based regression analysis is

$$L(i, t_{i9}) = \alpha + \beta T(i) + \gamma P(i) + \delta T(i)P(i) + [V(i)\boldsymbol{\delta}] + \varepsilon \quad (7)$$

where $P(i)$ is the propensity score treated as a continuous covariate and $T(i)$ the treatment indicator for treatment prior to last visit and $T(i)P(i)$ the interaction term.

References

1. Hernán, M. A., Brumback, B. and Robins, J. M. (2001) Marginal Structural Models to Estimate the Joint Causal Effect of Nonrandomized Treatments. *Journal of the American Statistical Association*, **96**, 454, 440-448.

2. Estimating the causal effect of zidovudine on CD4 count with a marginal structural model for repeated measures. *Statistics in Medicine*, **21**, 1689-1709.

3. Marginal Structural Models to Estimate the Causal Effect of Zidovudine on the Survival of HIV-Positive Men. *Epidemiology*, **11**, 5, 561-570.

4. Marginal Structural Models and Causal Inference in Epidemiology. *Epidemiology*, **11**, 5, 550-560.
